# The value of peer-support after the 22 July 2011 terrorattack: a follow-up focus-group study of gatherings for survivors from Utøya in Tromsø, Norway

**DOI:** 10.1186/s40359-026-04921-8

**Published:** 2026-06-04

**Authors:** Eirik Mathiesen, Lisbeth Straumsnes, Tore Sørlie, Martin Bystad, Rolf Wynn

**Affiliations:** 1 Division of Mental Health and Substance Use, University Hospital of North Norway, Tromsø, Norway; 2 Department of Health and Care Services, Tromsø Municipality, Tromsø, Norway; 3 Department of Clinical Medicine, UiT, the Arctic University of Norway, Tromsø, Norway; 4 Department of Clinical Medicine, University Miguel Hernandez, Alicante, Spain

**Keywords:** Terrorism, Trauma, Posttraumatic stress, Community, Group processes, Peer support groups

## Abstract

**Background:**

This article addresses the significance of group-based follow-up for a group of young survivors (*n* = 20) from Tromsø after the 22 July 2011 terror attack on Utøya in Norway, where 69 people were killed. The group was started at the request of the survivors and took place for two hours every other week, totaling 64 gatherings, during the period 2011–2014. It was led by two experienced psychiatric nurses and consisted of one part where the leaders introduced psychoeducational topics and another part with free reflection focusing on the emotional challenges the participants wanted to discuss.

**Materials and methods:**

The informants (13) were gathered for focus group interviews in 2023, 12 years after the terror event. The interviews were recorded on video, transcribed, and the analysis was conducted as an interpretive, thematic qualitative analysis, inspired by principles from Grounded Theory. All the informants were given the option to review the draft of this text.

**Results:**

The participants expressed that they greatly benefited from the group sessions and that the sense of community within the group was the most important aspect, still representing a central belonging in their lives. They emphasized that knowing others who understood the traumatic events and experiences made it possible, over time, to endure and process memories well enough to move forward in life in a positive way. A clear finding was the importance of starting early and maintaining a “watchful waiting” approach for as long as required for the traumatic experiences to be processed. At the time of the follow-up study, this process was still ongoing for all participants, even though the greatest need for help had passed when the groups were discontinued after three years in 2014.

**Discussion:**

The participants were generally very positive to the group sessions. They experienced that the group fostered a sense of community and belonging. It was a crucial element of these gatherings that professionals took responsibility for establishing and leading the group, while also emphasizing factors such as psychoeducation, normalization, humor, activity, and solidarity. Over time, it is important to work towards survivors generating a sense of belonging and agency that can help them face future challenges.

**Supplementary Information:**

The online version contains supplementary material available at 10.1186/s40359-026-04921-8.

## Background

On the afternoon of 22 July 2011, Norway was struck by two consecutive terror attacks of unprecedented scale since World War I I [1]. First, a car bomb exploded in the government quarter in Oslo, killing eight people and wounding many more. Later that day, the perpetrator carried out a mass shooting at the summer camp of AUF (the youth organization of the Norwegian Labour Party) on Utøya, a 120-acre island in Tyrifjorden in South-East Norway. Participants came from municipalities across the country. At the time of the attack, there were 564 people on Utøya, and 69 people (33 minors) lost their lives. In addition, 68 people required hospital treatment for physical injuries, many over an extended period. The event was quickly recognized as a national catastrophe [2].

The perpetrator had published an extensive manifesto online that same day, making it clear that he was motivated by far-right, xenophobic ideas. He aimed to target the social democratic movement, particularly the younger generation of politicians (AUF), whom he blamed for promoting a multi-ethnic society in Norway. The terror attack thus immediately acquired a clear political dimension [3].

The health authorities quickly decided that the responsibility for the survivors’ follow-up would lie with crisis teams in the various municipalities where the survivors resided while the responsibility for the bereaved would be based at the Center for Crisis Psychology in Bergen [2, 4, 5]. The recommendation was that all survivors should have a contact person from the healthcare system to help coordinate follow-up. A central principle was that the assistance should be proactive. Those who encountered survivors were also to conduct a simple assessment of their mental health. The Directorate of Health also recommended that municipalities hold gatherings for survivors and their families in the months following 22 July, where all types of questions could be addressed after returning home from Utøya [5].

In the week following the attack, a formal collaboration was established at the leadership level between the municipality of Tromsø and the specialist health services, and individuals responsible for continuing the work were appointed. The next phase involved mapping and establishing contact with the affected survivors through contact people, as recommended by the Directorate of Health [5]. Survivors were advised to have a low threshold for contacting their general practitioner, and in the weeks and months that followed, many were referred for treatment in specialist health services and with psychologists at other institutions. The youngest were received by a team of professionals at the Child and Adolescent Psychiatric Services, where a family-based approach was established. The work was followed up administratively throughout the autumn, and a professional team consisting of representatives from Child and Adolescent Psychiatric Services, Adult Psychiatric Services, and Tromsø Municipality was quickly formed. This team discussed how to address clinical challenges that arose in the work.

During the autumn of 2011 and spring of 2012, several gatherings were held for survivors and their families [6]. At one gathering in early autumn 2011, one of the youths expressed a desire to establish a group exclusively for survivors. This was based on the positive experience they had at Sundvollen (a hotel located near the Utøya island, used as a base by survivors, next-of-kin, emergency services, press, etc., following the terror attack), where they met other survivors to talk about what they had experienced. The proposal was discussed with leaders, experienced clinicians, and specialists from the Regional Resource Centre for Violence, Traumatic Stress, and Suicide Prevention, after which the first gathering was held in September 2011. Gatherings were then held regularly for two hours in the afternoon every other week, and by the time they ended in the summer of 2014, 64 gatherings had been held for the survivors from Tromsø (one person who had moved from another municipality joined later) [6].

The follow-up in Tromsø also included individual therapy with therapists in various parts of the municipality, but information regarding individual treatment was not collected for the present study. The primary focus of this study is the content of the group sessions and the experiences of those who participated in the group sessions from 2011 to 2014.

### How the group started

When the group first met in autumn 2011, the group leaders (the first and second author) had few clear ideas about the goals, content, and format of the gatherings beyond simply meeting. The survivors likely had no clear ideas either. However, their shared need and desire to be together was unanimously expressed. In a situation where they knew little or nothing about what lay ahead, it seemed intuitively right for them. And when people gather after such an event, it was natural to ensure good food and a safe, comfortable place to be. As leaders, we had limited experience with group therapy but extensive experience in meeting individuals and families in crisis after injuries and sudden deaths. We also had training in organizing relief efforts after natural disasters and accidents. However, the July 22 incident was in many ways different: it was a politically motivated act of terrorism and affected both nationally and locally on a large scale [1]. Being a man-made disaster made the task more complex and required us to seek new approaches.

Although the gatherings shared some features with group therapy, they were not intended as formal therapeutic interventions. The emphasis was rather on creating a structured, but also flexible space where participants could meet as peers, supported by professionals who contributed with psychoeducation and discussions when needed.

The first gatherings were relatively unstructured, and the youngsters spoke freely about what was on their minds now [6]. This was a time of intense media pressure and individual stories about what had happened on Utøya were constantly emerging. Several participants were also interviewed themselves [7]. Some were in hospitals, and others had begun therapy for their psychological reactions. Messages on social media from across the country were often strong and emotional. The impressions and questions were many, and the youths needed to share. Gradually, recognition emerged of what the survivors struggled with most and needed to be discussed more systematically. Initially, almost everyone participated, and it quickly became clear that we needed to divide into smaller discussion groups (6–7 per group) and have a common program for the topics to be addressed. Topics included sleep, school, family, romantic relationships, friends, substance use, later crisis reactions, trauma, and post- traumatic growth [6]. Additionally, they discussed thoughts and feelings about new terror attacks and other disasters that occurred in the years following 2011. The rationale behind the choice of topics was a combination of what we as professionals deemed important and what the youths themselves brought up. In addition to the two permanent leaders, other staff were also involved in the first year to talk about requested topics. It functioned as an ongoing psychoeducational-like group.

It is important to emphasize that the format evolved because of ongoing discussions with the youths about what they experienced as most important for them. As leaders we had no standardized approach to rely on. We had to create something “new,” and no one could know in advance what it would look like. This is important to distinguish from what we later read about experiences with peer support groups elsewhere. This was a group of youngsters with a considerable degree of self-confidence and organizational competence and experience. At the same time, it was important to remember that they were severely traumatized and had a need to be cared for. Initially, it was about sensing how everyone was doing. The pressure to speak freely and mix in anecdotes and humor was often great and had to have its place. Nevertheless, the youths recognized the value of maintaining a certain focus for a period. Gradually, it became customary for us as leaders to introduce a psychologically relevant topic for discussion for about half the time. This was followed by a break with food and drink, and then free discussion for the remainder of the time. The free part had value beyond serving as “bonding” and socialization, as often “guards” were down, and sometimes very important realizations and conversations emerged, where we as leaders had to be attentive and tie the threads together when needed. Perhaps it concerned a significant event in the family or how one participant reacted to a new terror event in the media that they wanted to share.

Over time, the conversations revolved around many of the same questions but gradually in a deeper and more trusting way [6]. People got to know each other better and developed the ability to be open and candid. The survivors clearly had a need to just talk-talk repeatedly-to someone who knew how it was and had been: to release a kind of pressure in a space that felt safe and was immediately understood. The gatherings lasted two hours and were held every other Thursday afternoon in a fixed location with a homely atmosphere. This frequency proved sufficient to keep up with developments both in the “case of 22 July” and for the individual, and to become aware in time if something was not working, e.g., at school. The community was also a place where one could express concern if a survivor had not attended for a while: did anyone know, perhaps someone could send a text message to check how they were doing?

### Aid work in international terror incidents

Initially, there is often much chaos and difficulty organizing adequate and sufficient psychological help after large-scale terror incidents or similar events, such as school shootings. Some of the challenges involve insufficient guidelines, lack of oversight, long waiting times, bureaucratic resistance, insufficient funding, and difficulties in securing qualified therapists at the right time and place [8]. This applies to both individual and group interventions for survivors. When it comes to groups, certain organizational measures and logistical resources are additionally required.

There is limited research regarding the use of groups in follow-up after terror incidents [9, 10]. One relevant example is the follow-up after the school shooting in the Finnish town of Kauhajoki in 2008 [11, 12]. There, an extensive program was conducted over several years for bereaved families, surviving students, and others affected in the local community. They emphasized the importance of continuing the work long enough and highlighted the principle of “watchful waiting” [13]. A central element in the follow-up by the responsible team was peer support groups led by professionals. The program lasted two years and consisted of five weekend gatherings based on elements of psychoeducation and group work. In their work, it was important to start groups at the right time based on knowledge of where the affected were in their grief process. They also emphasized psychoeducation and identifying signs of post-traumatic reactions and the stage of grief. It was also vital to identify the unique aspects of the grief process in each family, establish attachment-oriented elements in the group process, and strengthen the cohesion of the families’ natural networks by encouraging belonging and sharing of feelings [12].

Another example [8] is the group that started four months after the terror incident at the Bataclan club in Paris in 2015, for survivors from England. Interested and affected psychotherapists organized 10 gatherings, each lasting 2.5 h, over a year. They summarized that the group was useful in overcoming isolation and establishing mutual understanding and emotional support. For the survivors, it was about having the opportunity to meet others in the same situation and not being alone with their experiences. It was found that meeting face-to-face was more important than only connecting via social media. In the group, they could make sense of what had happened and together figure out the “puzzle” that arose in the chaos after the incident. The gatherings were also important for feeling less lonely and thus overcoming isolation. They also fulfilled a need for information about rights, insurance, legal processes, etc., in addition to how to handle the media and emotions related to the anniversaries of the attack. The author reported that many participants stated that the group had been a “lifeline” for them [8].

A third relevant example is the work initiated after the terror attack in Manchester in 2017, where peer groups were a central part of the follow-up. Initially, they had contact with about 100 people who showed interest in participating in collective programs, and eventually, 15 groups were established. The purpose was also to give survivors and bereaved individuals the opportunity to share thoughts, feelings, and experiences collectively and benefit from psychoeducation. Eyre [9, p. 11] states: “The results suggest that facilitated peer support has enabled bereaved people, survivors, and responders to share and make sense of their experiences, benefit from mutual support, and enhance their coping and resilience”.

Research on psychosocial responses to terrorism and mass trauma highlights that recovery is not only an individual process, but also unfolds within social and relational contexts. Studies of collective trauma emphasize how shared exposure to extreme events can affect both identity, meaning-making and long-term adaptation [14]. In addition, the social identity theory suggest that shared experiences of threat may strengthen in-group identification and create boundaries toward others who have not had the same experiences [15]. Recovery-oriented approaches further underline that adaptation after trauma is often non-linear, prolonged, and supported by both individual and collective resources [16, 17].

While there is a growing body of research on peer support and group-based interventions following extreme events, several core processes (e.g. shared understanding, normalization, and reduced isolation) are already well documented. The present study aims to describe how these processes developed over time in a psychoeducational-like group, led by professionals, that started early and continued for several years.

In the present study, we wanted to explore how survivors from Tromsø municipality experienced the group gatherings over time, and how these experiences can be understood in light of perspectives on collective trauma, social identity processes, and recovery following extreme events .

## Materials and methods

### The participants

Of the approximately 20 survivors who regularly participated in the gatherings from 2011 to 2014, 13 participated in the focus group interviews in 2023 (about 65%). There were seven women and six men, reflecting the gender distribution in the original group. The average age at the time of the interviews was 28.7 years. A small majority (7 of 13) had partners. Three of the informants also had children. All reported being in some form of work or study. Most described significant delays and challenges in their educational paths. Several had completed university-level education, but there was still considerable variation in how resolved they were regarding their choice and completion of education. Some informants had been followed up by the healthcare system for years due to severe physical injuries, and the impression was that all had tried various forms of individual mental health therapy, but this information was not gathered systematically.

### The focus group interviews

The 13 participants, who came from across the country, were gathered during the weekend of 23–26 February 2023 in the city of Tromsø. They were received by the same individuals who had led the group on the same premises used for the group gatherings. Detailed information was provided about the plans for data collection and the preparations to care for individuals. A social framework was established, and participants were divided into two groups. A total of four focus group interviews, each lasting about 90 min, were conducted over two days. The interview guide formed the point of departure for the interviews (Additional file 1). However, relevant topics not covered by the guide also emerged and were discussed during the interviews. The interviews were audio and video-recorded, and participants also completed a short- written survey (data not included here).

The primary reason we chose the focus group design was our belief that the interaction during the interviews would elicit a maximum amount of information—information that likely would not have emerged in one-to-one interviews. That the design also mirrored the dynamics of the original group was secondary, though still relevant. An additional factor was that the participants supported one another in invaluable ways when sensitive topics arose during the interviews.

The focus group interviews were conducted in 2023, 12 years after Utøya. Previously, a summary report was written in 2014 (LS), and an article was published in 2015 (EM). This material was available to the entire research group.

Under the section on “limitations,” it is noted as a weakness that no systematic log was kept during the period 2011–2014, meaning that in practice both the informants and the interviewers had to rely largely on memory. As stated, the main body of the material is based on what the informants expressed in the present day (2023) about how they experienced the events during 2011–2014 and 2014–2023. The research project must therefore be regarded as a retrospective study.

This can be divided into two aspects. In the focus group interviews, the informants primarily expressed what they currently (2023) thought about what had happened, but they also reflected on who they were and what they had thought during the period 2011–2014, as well as in the years afterward.

Thus, the different temporal perspectives repeatedly became an important topic during the interviews and a source of valuable information. For example, this concerned the contrast between a teenage perspective (2011) and a (young) adult perspective (2023). This was despite the fact that memory varied, likely for trauma-related reasons. The retrospective perspective in the interviews also strengthens the likelihood that the informants expressed themselves from a position in which each individual had considerable time to reflect on their experiences.

### Analysis

The analysis was conducted as a thematic qualitative analysis, informed by principles from Grounded Theory by Charmaz [18]. The process began with careful preparation and conduct of the focus group interviews, where attention was paid to participants’ statements, emotional expressions, and interactions. The interviews were transcribed by the researchers through repeated listening to the recordings, which facilitated a thorough familiarization with the material.

The analysis involved close reading of the transcripts, coding of meaningful segments, and grouping of codes into broader themes. Through this process, patterns and connections across participants’ accounts were identified. Themes were developed by comparing across interviews and discussing them among the authors.

The analysis was primarily grounded in the interview material, while the authors’ prior clinical experience was used as background for interpretation. Given the authors’ dual role, extra attention was paid to distinguishing between participants’ prior experiences, and to critically reflecting on how this involvement might influence the analysis.

From the very beginning of the research process, we were aware of the risk of bias. We therefore had to reflect on whether it was at all possible for us, as former clinicians, to carry out this research. The issue of bias is inherent in any situation where clinicians seek to investigate their own work. The most obvious response was to involve competent and independent co-researchers, and to take the issue of bias with the utmost seriousness both in the data collection itself and in the analytical and descriptive processes.

The most important aspect was to remain conscious that our task as researchers was to discover how the process had actually been experienced from the informants’ perspectives (both during 2011–2014 and in 2023), even when these perspectives differed from our prior understanding or later hopes. Furthermore, we had to be prepared to acknowledge that some of our hypotheses had not proven correct, and that we had made mistakes deserving of criticism.

The discussions within the research group—both in developing the interview guide and in interpreting the transcripts—helped provide new perspectives and counterarguments regarding how the informants had experienced the group. We were also aware that parts of our prior understanding (as clinicians) could be flawed or insufficient, and influenced by factors such as time pressure and stress. This was discussed both within the research group and with the informants during the focus group interviews, resulting in important corrections.

Another important factor was that nine years had passed since the follow-up ended, which in itself had created a certain degree of distance. During this period, the survivors had encountered many other therapists and healthcare providers. Thus, although the immediate follow-up period (2011–2014) had undoubtedly been life-defining, life had moved forward, and the young adults had developed new perspectives. To ensure an independent perspective, we also consulted an experienced colleague in trauma treatment during the process, which resulted in an increased focus on the issue of potential bias.

Because the authors had a dual role as both clinicians and researchers, particular attention was given to reflexivity throughout the study. The analysis was primarily based on the interview material, and we sought to distinguish clearly between participants’ accounts and our own prior experiences.

To reduce the risk of confirmatory interpretation, the analysis involved repeated discussions among the authors, where alternative interpretations were considered and challenged. We also paid attention to variations and divergences in participants’ accounts, rather than focusing only on common themes.

The authors’ prior involvement was considered both a potential source of bias and a resource for contextual understanding, and this challenge was addressed through ongoing reflexive discussion during the analytic process.

### Theoretical perspective

As one analytical framework, we draw on the concept of Sense of Community as described by McMillan and Chavis [19], particularly to illuminate processes of belonging, cohesion, and mutual influence within the group. Their definition of Sense of Community is based on a feeling of belonging, that members matter to each other and the group, and a sense that needs will be met through the commitment to being together. They describe the elements and dynamics of how cohesion in groups is created and leads to a sense of community.

According to McMillan and Chavis [19], some elements are of particular importance relating to the concept of Sense of community. One such element is that of membership, which means identifying with the group and having invested in part of oneself to become a member. This also involves rights and obligations, emotional safety, and a shared symbolic system characterizing the group is emphasized, contributing to maintaining the group’s boundaries. A second element is the individual’s ability to influence development and the group’s influence over the individual. Third, participation in the group must involve reinforcement and satisfaction of important needs for the members, and success in fulfilling these, including shared core values. A fourth element is that of emotional connection, which is typically linked to shared history. A final point is the importance of sharing common spiritual values [19].

The authors outline how the various elements influence each other in a self-reinforcing and circular way, so they are both cause and effect. Boundaries ensure the protection of intimacy [19]. The emotional safety resulting from secure boundaries leads members to recognize that there is a place for them in the group and that they belong. A sense of belonging and identification facilitates the development of a shared symbolic system, which in turn defines the group’s boundaries. Likely, feelings of belonging and emotional safety lead to “self-investment” in the group, which in turn gives the individual a sense of deserving membership. Regarding influence, when the group influences the member, the person can also have more influence in the group. If, on the other hand, one opposes the group or tries to dominate it, this leads to less influence. The authors argue that if people voluntarily choose whether to conform, their need for consensual validation strengthens the group’s norms. Reinforcement at the group level allows people to be together in a way that fulfills everyone’s needs [19].

### Approvals and ethical considerations

The study was approved by the Health Research Ethics Committee of North Norway (REK North 427996) and by the Data Protection Officer of the University Hospital of North Norway (PVO 2979). All participants gave written informed consent. When the study planning began (2020), it was clear that ethical considerations and privacy issues had to be carefully addressed. The relatively small number of informants in a compact urban community, where there had been significant media exposure at times, posed challenges regarding the risk of identification. However, the most important reason was that our informants had suffered great losses and severe trauma, and the risk of re-traumatization could not be ignored. The first issue was resolved by the researchers committing to thorough anonymization, and the informants were given the opportunity to review the text before publication. Regarding the second issue, the researchers acknowledged that strong emotions could be triggered during the interviews. However, the informants knew each other and the researchers well from before, and this risk was largely offset by the need to meet again and share how their lives had developed. Nevertheless, it was agreed that during the focus group interviews, the possibility of reactivating highly distressing reactions had to be considered. Therefore, it was decided to have healthcare personnel (doctors, nurses) available to provide help and support if needed and, if necessary, ensure referral for further treatment. Despite strong emotions being expressed during the process, it was not necessary to involve healthcare personnel at the time. This was likely because their presence provided a framework of safety, and the informants themselves, within familiar settings and with leaders they trusted, took good care of each other during the interviews and breaks.

A central research ethics question was how to address the fact that the focus group interviewers (2023) were the same clinicians who led the gatherings from 2011 to 2014. Initially, neutrality and objectivity are important factors in all research, and when we began the study, we were aware of this challenge. It is undoubtedly true that we were important to the clients at the time. However, we argue that the advantages of familiarity with the group, a good understanding of the situation, and the ability to use a method that mirrored the conversations from 2011 to 2014 outweighed the disadvantages regarding neutrality.

After the group interviews, the interviewers discussed passages where they had experienced reduced presence in the participants’ communication and how their preconceptions based on their earlier therapeutic role might have influenced the process.

When we gathered the informants before the focus group interviews, it was emphasized that the purpose in this context was scientific. Furthermore, we clarified that we sought the most honest statements from their side and that any loyalty bonds should not prevent them from making critical remarks. It was thus made clear that the goal was primarily to obtain the most truthful impressions, and that therapeutic priorities could not take precedence beyond general compassion and attentiveness during the interviews.

## Results

The most striking finding was the strong sense of solidarity among the survivors. We will discuss the foundation of this community and describe how intuitive mutual understanding developed. Another central finding was the significance of time, both in terms of starting group gatherings early, understanding that issues expressed early could re-emerge later, and how the perception of time could vary for individuals and between people. In the second part on organization, we will discuss participants’ perceived importance of leadership, structure, availability, and the benefits of sharing practical advice with each other. The final part addresses how significant it was for survivors to explore what was normal together and to allow space for humor. We will also describe how group processes developed and discuss how survivors experienced the relationship between individual and collective follow-up.

Based on the analysis of the data, we have created 3 main categories and 10 sub-categories. An overview of the main categories and sub-categories is presented in Table 1.

**Table 1:** Overview of categories

Main categories	Sub-categories
1) Community and the significance of time	1.1) Standing Together - An Intuitive Mutual Understanding
	1.2) The Perceived Significance of Community Over Time
2) Organization	2.1) The Importance of Structure
	2.2) The Need for a Dedicated Arena
	2.3) Low Threshold
	2.4) Sharing “Tools” and Practical Support
3) On the normality of psychological reactions	3.1) Normality
	3.2) Humor
	3.3) Group Processes
	3.4) Community in Gatherings Versus Individual Therapy

### Main category I : community and the significance of time

#### Subcategory 1: standing together – an intuitive mutual understanding

*“It was a terrible thing that brought us together*,* but we stayed together afterward.”*

*“Because it was and is very*,* very difficult to be alone.”*

*“But knowing there’s a group that just inherently knows that some days are tougher than others, especially in the early phase.”*

The gatherings were based on the participants’ shared experience of the Utøya attack, although their individual experiences were different. The participants also knew each other beforehand through political contexts. Participants emphasized that being together with others who had gone through the same extreme event was of major importance. They described a strong sense that only those who had been there could fully understand their experiences, even though others might try to empathize. As one participant expressed it:

*“(That) no one who hasn’t experienced what we experienced can understand the scope of it.”*

When you have each other after such an event, you feel safer and not alone-something that was clearly both a strong feeling and a reality. Initially, they were completely helpless and at the mercy of the situation. The community then became a shield against the gnawing feeling of being isolated with their fear forever:

*“… this is the worst thing we’ve experienced in life*,* and that’s why the community is particularly important.”*

In contrast to the strong sense of unity and togetherness they had with each other, several survivors described encounters with the outside world as not always easy to handle. They spoke of interactions with people who wanted to know more about the event and what had happened to them. They experienced this challenging, having to adjust and prepare their friends for something they knew the others could not be mentally prepared for, often resulting in a complete change in the atmosphere (e.g., at a social gathering). The survivors had repeatedly found themselves having to take care of the other person in a situation where their own inner state was still fragile. One survivor shared how they eventually learned to tell interested friends, “Now is not the time,” and instead choose a more suitable moment to share their story about 22 July. The same applied to some extent within their own families.

#### Subcategory 2: the perceived significance of community over time

*“And also*,* that community isn’t something you feel you need at the time*,* but in retrospect*,* we see how important it was. At the time*,* you might not think*,* ‘I need to be with someone.’ That was so important. In hindsight*,* as several have said*,* we created it together*,* and it has grown stronger over the years.”*

*“And I think the community became stronger because we also had an organized community. Where we got help to push the right buttons. Which you didn’t necessarily realize you needed to push. …but that community was there as a foundation you could step in and out of… It was an important foundation that you knew you had without having to use it all the time.”*

*“…that how things have gone hasn’t been like a graph going in one direction. It’s gone up and down a lot*,* and it hasn’t been the same for everyone. People have had really good periods*,* and then not so good periods*,* and that’s also completely normal.”*

The statements highlight that the experience of community among the survivors was crucial in the beginning and remains so, even though its form has changed. The fact that the significance of the community changed over time was undoubtedly both inevitable and necessary. And that the development was dynamic in the sense that they constantly had to adapt to new realities. Several participants pointed out that within the group, they had a different sense of time than others, in the sense that they had a clear perception that most people, including well-informed classmates, had moved on or were in the process of doing so. While many described this, others emphasized different experiences over time.Therefore, it was so important to be with those who experienced that “we are not done with this,” and that the time for finding security and inner peace had not yet arrived. Survivors expressed that it was difficult during the process not knowing how long the group could continue, fearing that everything might fall apart if the group had to end for some reason. They recognized early on that the Utøya experience would stay with them forever, so the question was more about how they could manage it. It was comforting to have someone you could truly compare yourself to—not to walk in step, but perhaps to strive toward or find solace with because the same problem (e.g., sleep difficulties or lack of concentration) recurred month after month. In this way, it was important to have a shared experience of being on a timeline where inadequacies (like not being able to get out of bed in the morning) were still a reality. And that even small progress (“achievements”) like getting out of the house was given significance others could not imagine. To emphasize the sense of value and gratitude, several members expressed compassion for those who did not receive such an offer, i.e., in other parts of the country.

### Main category II: organization

#### Subcategory 1: the importance of structure

*“That it was organized. So*,* there was security. If something came up that we couldn’t handle*,* there was someone who could handle it. Someone we could trust.”*

Several survivors pointed out the importance of the gatherings being organized and led by healthcare professionals. This meant they were held at regular intervals and followed a pattern over two hours, divided by a break with a meal. Although the food was usually simple (buns and soda), it likely played a not insignificant and homely role: being given something while also easing tension.

*“There’s something about that sense of community-sitting and eating together -and just having a good time together- also. To contribute to creating a situation where it becomes natural to talk about things.”*

Participants described that the structure created predictability, making it clear who was in charge. The youths knew that they had gone through something serious, where during the attack, they had to try to take responsibility for each other under very demanding circumstances. But they still lacked the words to explain what they had been through, so probably it was suitable that adult professionals oversaw the gatherings, which made the youths feel safe and able to relax.

#### Subcategory 2: the need for a dedicated arena

*“We had (previously) a community through politics. We needed an arena where we could discuss Utøya and have that as an agenda. So that (when) we have the community tied to our political standpoint or organization*,* we can focus on that there. In the political arena*,* not everyone takes part in our Utøya community. “*.

Several survivors knew each other beforehand, primarily through political work. And when they returned home from Utøya in late summer 2011, the election campaign was just around the corner. Many threw themselves into the work and met each other there. The question could be raised whether this was not a sufficient meeting point for the survivors. However, there were several factors against such a notion. Firstly, not all survivors were equally engaged in political work. Moreover, not all survivors participated in AUF, for example, in the 2011 election campaign. More importantly, politics had its own dynamics, where there was no room to address what had happened on Utøya. At the gatherings, they could “take a break” from politics and just be together with one goal in mind: to help each other move forward in life after the trauma.

Early on, there was uncertainty about whether others besides the survivors could participate because they were friends and affected. It was quickly clarified that this was not appropriate because the reactions were very different and that friends needed to have their legitimate needs met in another way.

#### Subcategory 3: low threshold

*“You can come as you are*,* and at the stage you’re at. You can say a lot or a little*,* and everything is okay.”*

Several survivors emphasized that it was nice that it was easy to show up and that even though they cared about each other and might wonder if someone did not show up, there was no obligation. It probably would have reminded them too much of school, where obligations naturally play a significant role, and where rules about a certain number of allowed absences were highly debated. One participant said that the fact that it was fun to be at the gatherings made the threshold for going lower. Another stated that it flowed a bit more easily than if it had just been problem-focused group therapy and “heavy” and that it would have taken more energy than it gave. Several had experience with trauma therapy from individual sessions and emphasized that in the group, it was not supposed to be like that, but rather “just being together,” where they could meet to talk about Utoya, but not necessarily in a very serious way, as one put it.

#### Subcategory 4: sharing tools and practical support

*“I think that when it comes to the community*,* we were concerned with the same things. We shared tools and tricks with each other: To help each other.”*

The conversations among the survivors were about everything and largely about how they were doing in their daily lives. It could be about sleep difficulties or problems at school, e.g., with absences or lack of concentration in studying. Then one could share how they struggled with the same or how they managed to overcome a problem. It became easier both to identify and strive toward solutions. After “Utøya,” there were also a wealth of practical challenges. From how to get healthcare to how to handle insurance matters. At the gatherings, they could inform each other about the solutions some had found, either on their own or through their families. Including the leaders, there was also a good deal of competence in the families that was shared when the youths met. In this way, the gatherings also became a place where one could find answers, perhaps not immediately, but eventually find a solution to both trauma- related challenges and practical questions. The gatherings were also a place where one picked up information about each other, for example, if someone was sick or something had happened in the family, and they did not show up. Then someone would often take on the responsibility of checking if there was something the rest of the group could contribute to, or if the person just needed a «nudge» or a sign that they were missed-and welcome back.

### Main category III: on the normality of psychological reactions

#### Subcategory 1: normality

*“I also think what was good for us was feeling that everything was normal… I remember we joked a lot about how if you do this and that*,* it’s normal… What happened is terrible*,* can I be happy? But you see that everyone else manages it too-and that’s a good feeling. So*,* you felt that it was allowed and good*,* and that all the other feelings were also somehow allowed and normal. So*,* I think those were the topics that came up*,* like sleep. Everyone had trouble sleeping. Many had trouble with the same things and could understand each other on a completely different level.”*

In a situation where the survivors experienced many feelings that were new and unusual to them, being able to talk to each other about what was normal was important. They had probably heard it from therapists and other healthcare professionals, but when the thoughts and reactions became too unusual and the fear of being abnormal or ‘crazy’ took over, it was comforting to notice that the others felt the same way. Then they realized that what they themselves were experiencing was not so unusual, and they could relax. Several also pointed out that it was nice to have “a lot of leeway,” both in terms of behavior and statements. As one put it:

*“And of course*,* if someone starts crying*,* gets drunk*,* or starts laughing hysterically after a funeral-no one has been upset about it. We’ve stood together and allowed everyone to have the reactions they have. It’s been much easier to have the reactions that come naturally. Without hiding it or being afraid to show it.”*

Recognizing feelings, they might initially have thought were very special, but then saw the same in others, expanding their own concept of normality. This became an internal reassuring joke, that ‘everything is normal.’

#### Subcategory 2: humor

*“So I think that the fact that i t was also fun to be there made the threshold for going lower. So*,* the connection between serious and humorous works better then.”*

The survivors emphasized that it was generally fun to be at the gatherings. That was something they looked forward to. And that they were met by others who “cheered” for them and greeted them with encouragement. In addition to a “serious” part with defined psychological topics, they could joke and laugh. Humor thus served an important function. It likely created a balance and counterforce in an otherwise harsh reality. The youngsters were aware that the internal humor could be quite dark and that it only fit there: no one else could say the same, and no one else could hear it. The sometimes-macabre humor perhaps also allowed them to express feelings that would otherwise have been difficult to put into words.

An occasionally lighthearted atmosphere made them more comfortable with each other, something that defused the situation. They were also ready to take on the challenge when conversations could swing between the humorous and the serious in an effortless way. This could not be planned and had to develop naturally when it occurred. Nevertheless, it was important to be attentive and aware. We believe some of the most important and profound conversations arose in such situations. Perhaps this was because they developed naturally and with low tension because the conversations arose without planning—when the time was right.

#### Subcategory 3: group processes

*“On the other hand*,* I also think that you became aware: ‘Oh*,* I shouldn’t feel like this. ‘At least I often felt: ‘Oh*,* he manages this and that*,* and she manages that: what’s stopping me from managing it?’ I think that feeling was a tool to think*,* ‘I’m here now*,* but they’re three steps ahead of me.’ And there’s no reason they should have a ‘lead*,*’ so I think there was an element of healthy competition*,* for lack of a better way to express it. We pushed each other forward a bit too-because some achieved and managed things that others didn’t. Got past a feeling of anger or frustration.”*

The gatherings of survivors came about because the youths expressed a desire to be together in an organized way. As leaders and co-developers, we naturally hoped it would have a health-promoting effect, and although the gatherings were not intended as formal group therapy, it was still a group with dynamics, and thus a forum where group processes unfolded.

Despite not everyone attending every time, it was still the same group of people meeting each other for over three years. Then processes arose almost by themselves. Statements from individuals mostly flowed freely and were somewhat validated (empathy, acknowledgment) by the leaders, while also trying to stick to and deepen relevant topics that arose for individuals, such as school absences and family crises. In this way, a response developed among participants depending on how affected they were, sometimes based on empathy, but also reflections on how they themselves had tried to find a solution to the same problem.

Several expressed in the focus groups that they became aware of differences in the emotional range regarding others’ reactions and that they learned a lot from it. Even that the emergence of others’ feelings meant they themselves “didn’t have to” go into the same. Some spoke of progress they observed in others, which made them stretch themselves with the help of “healthy competition”, demonstrating that group processes were taking place.

#### Subcategory 4: community in gatherings versus individual therapy

*“This has been much more effective for my way of dealing with trauma than going to a psychologist and sitting alone talking about something the other person can’t relate to. Whereas in this room*,* you could say: ' I felt exactly the same.”*

Many participants described the group as more helpful than individual therapy in the early phase, although experiences varied. The impression was that many had tried individual solutions but did not find them sufficient. They found it easier to figure things out when there were several with the same experience and not dependent on whether they could get the therapist to understand what they had been through.

Some also experienced that therapists did not seem emotionally prepared for the realities of what they had experienced. Some also pointed out that therapy often became very heavy and that they needed an arena where they could alternate between seriousness and joking.

## Discussion

In this study, three findings proved to be decisive. The most essential was the survivors’ sense of community based on shared experiences, both before, during, and after the attack on Utøya [19]. Starting follow-up early and continuing as long as necessary was also very important [11, 12]. The structured and flexible organization of the group was also of great significance in creating safety and normalizing reactions to the trauma, thereby creating a basis for mutual support among the survivors [17]. Taken together, these elements illustrate how the establishment of peer support groups led by professionals may be experienced as helpful for survivors to manage life in the long term [10].

The intervention differs from traditional group therapy because it was not based on a predefined therapeutic protocol. At the same time, it goes beyond informal peer support by including professional facilitation and psychoeducational elements. It may therefore be best understood as a form of facilitated peer support.

Our contribution lies in its longitudinal perspective. The data from our study provides insight into a group that started early after the event and sustained over several years, with participants reflecting on its significance more than a decade later. This offers a clearer understanding of how peer-support processes develops over time following a collective trauma.

When we began the research project, we (as clinicians/researchers) carried with us a certain pre-understanding and assumption that the survivors (within the group, and perhaps more broadly) had experienced some form of health benefit as a result of the follow-up. One reason for this assumption may have been the positive attention that the follow-up work in Tromsø received through media.

### A sense of community

The experiences of the survivors can be understood in light of the theory of “Sense of Community” [19]. It was of great importance that they themselves took the initiative to establish the group, and continuously participated in shaping it, including decisions about how long it should last. This, in turn, led to a strong sense of ownership. Early in the process, attempts to include interested friends who had not themselves experienced the attack proved inappropriate, and it became clear that only those with shared experience from Utøya should be part of the group.

Another, and painful, experience was the difficulty of sharing what they had gone through with others who were interested. The atmosphere would change completely, and it often ended with them having to take care of the other person. When they were together in the group, they could avoid seeing themselves as victims of terror.

The survivors regarded the group as their most important community. They took care of each other, for example by checking in if someone was absent. They looked forward to meeting and emphasized that it was enjoyable to participate. The gatherings should not be too heavy, and spontaneous humor played a major role in easing the atmosphere.

The fact that the meetings were held regularly over time contributed to developing depth in the conversations. The strength of the community was based on a shared understanding of the background of the attack. This may have strengthened the sense of belonging within the group, while also shaping clearer boundaries toward those who had not shared these experiences. This in line with social identity theory, which suggests that shared experiences of threat can strengthen group identity [15].

Research within the “social identity approach to health” further suggests that shared group membership may function as a psychological resource in situations of crisis and adversity [20]. Identifying with others who have undergone similar experiences can strengthen perceived social support and resilience and reduce feelings of isolation.

### The importance of time

An important experience was starting early. The participants’ accounts suggest that recovery is not a straight-line process, but something that changes over time. At that time, grief, pain, and helplessness among the survivors were at their greatest, and they needed each other deeply. What happened in this phase was clearly important for recognizing the value of seeking help later—namely, that it was possible to accept help and find support in each other [11, 12].

The survivors also discovered that individual development could vary over time, and that some struggled longer than others to cope. They learned from each other and gradually understood that everyone needed to be patient, and that each person’s development had to take the time necessary. It thus became clear to the leaders that it was necessary to practice the principle of “watchful waiting,” not only in the period between 2011 and 2014, but also later [13]. This suggests a need for support systems that can adapt to different and changing recovery processes over time. Problems with “triggers” that created renewed feelings of insecurity could return at regular and irregular intervals. A key point was that they soon discovered that people outside the group—including leaders, family members, and helpers—often had a different sense of time, in that they relatively quickly considered themselves “finished” with “Utøya.” A common experience was that others seemed to move on more quickly, while the survivors felt that their own process took longer. Participants described this as surprising and experienced the group as a source of comfort, because there the processing could continue. A recurring problem, both in Norway and abroad, is that follow-up after terror attacks is ended too early [21–23]. How long the group should continue was therefore a central topic on which the participants had clear opinions.

The survivors realized that it took time to develop relationships with each other, and experienced that they became more trusting and open as time passed. It was not easy to know what was needed at the time, but retrospectively they recognized the value of having been together in the group over time—especially in establishing a sense of hope. Taken together, these findings highlight time as a key part of recovery after trauma, showing that support needs to be flexible and have sufficient follow-up time.

### Ownership, participation, cohesion, and structure

The survivors quickly developed a strong sense of ownership of the group. It became an arena where they could be themselves and with which they identified. This was naturally related to the fact that they had taken the initiative and requested support for establishing the group, but also that they shared responsibility when important decisions about direction had to be made throughout the process. This, in turn, created a sense of mastery through being involved in shaping the conditions for their own development.

At the same time, it was important that the leaders ensured that the framework was in place through organizing regular meetings and securing a comfortable and safe location. It was also important that participants, after everything that had happened, felt cared for by having healthcare personnel present in case things became difficult. The leaders were also important because they showed the survivors that adults other than their parents took responsibility for them in a situation where they partly felt let down by society.

From the leaders’ perspective, it appeared entirely necessary to take the initiative in developing some form of structure. After all, we had both a mandate from our organizations and a clear request from the young survivors to help create a constructive framework. This process, however, took place in consultation with the survivors themselves.

The relationship between participant influence and professional structure may be seen as a tension, and most participants were quite outspoken. There were, for example, differing opinions regarding how much time should be devoted to the various parts of the gatherings. There was also considerable disagreement regarding the timing of the group’s conclusion. Nevertheless, there was generally little overt conflict.

While participants described the group as shaped by their needs, the professional framework may also have influenced what was expressed and how experiences were understood. Leadership may have helped define what was seen as normal or as progress, which could both support participants and shape how experiences were expressed.

The gatherings were structured in a way that created predictability, in contrast to the chaos and instability the young people experienced in the initial period after July 22. The two parts of the meetings provided space both for organized discussion of important emotional themes (psychoeducation) and for free reflection, creating both structure and freedom. The shared meal midway through the meetings was something they looked forward to and helped create a good atmosphere in which it felt natural to be open with each other. All of this contributed to a sense of cohesion and a cheerful atmosphere that counterbalanced the serious background [17]. For the young participants, it was important that the gatherings were not too “serious” like (traditional) therapy, as that would become ‘too heavy’. Nevertheless, they could spontaneously introduce very serious topics during the free part. This freedom and flexibility was important.

### Significance of political background

What distinguished the attacks on Utøya and the Government Quarter was that the perpetrator explicitly stated that, in addition to targeting the government apparatus, the terrorist intended to strike at the future generation of political candidates within social democracy (the Labour Party and AUF). The terrorist attacks thus violently intervened in an already ongoing political struggle, both nationally and internationally, concerning immigration, refugees, and multiculturalism.

In the political debate that followed in the aftermath of the attacks, differing analyses emerged, and those who had promoted comparable—though nonviolent—political messages largely “washed their hands” of responsibility. For many of the young camp participants, this felt unreasonable, because to them it was clear that the hatred (toward people of color, immigrants, and refugees) had already existed long before the attack.

The sitting social democratic government chose to focus on unity and reconciliation, expressed through the massive “rose marches” marked by compassion and grief. There were undoubtedly reasons for adopting this approach, but despair and anger toward those who had “added fuel to the fire” were also present and, in a sense, were not accepted.

The young survivors felt deeply affected by the lack of clear political acknowledgment and support following the attack. At the same time as they struggled with severe trauma and everyday functioning, many experienced it as a heavy burden that they themselves were expected to carry the political discussion surrounding the injustice they had experienced.

That the political backdrop would have consequences for the processing of trauma was not surprising. The fact that few stood up for them likely did little to heal their wounds.

### Individual experiences in a collective context

All the survivors were united because they were affected by the same trauma, even though the specific events during the terror attack differed. Some were seriously physically injured and survived thanks to intensive medical treatment. Each participant’s psychological reaction was naturally also different, since they were individuals with different backgrounds and conditions.

In the follow-up of the survivors from Tromsø, many supportive resources and professional efforts were mobilized. It became a kind of collective effort in which many were willing to contribute, both because the situation was given high priority for obvious reasons and because proactive intervention was strongly emphasized by the central authorities.

All of this took place in addition to the collective gatherings organized through cooperation between personnel from the municipality and the specialist health services. In this way, a division of labor emerged between those working with individual interventions, those working with families, and those working with the collective group. Over time, a number of additional independent therapeutic-like projects were also carried out.

It is therefore important to emphasize that it was the combined effort that produced results. One lesson from the Utøya tragedy is thus that a broad mobilization can be necessary, and that interventions could take place on multiple levels simultaneously.

It was therefore necessary to have both individual and collective approaches. Experiences with individual therapy varied among the survivors. Several found it difficult to make therapists understand what they had been through. It could also seem that some therapists were not emotionally prepared for the brutal realities they had experienced. Others had positive experiences with individual sessions, which may indicate that these therapists had access to better-developed collegial support and perhaps a more developed system of care. Another issue may be that the young people early in the process did not have many words for what they had been through, and that the language used in individual therapy felt too unfamiliar. As time passed and the young people grew older, several expressed greater benefit from individual treatment.

In the group, they found resonance with each other and could use everyday language, which helped them experience coherence in life [24]. The group not only provided support, but also helped participants develop shared ways of understanding their reactions and coping over time. Through interacting with each other, they came to see what was normal reactions to trauma. In this way, the group also shaped how they made sense of their experiences. This is in accordance with research showing that group norms can shape how individuals interpret experiences and respond to others [25, 26].

Participants described humor as important in relieving tension and helped the survivors relax, also when new challenges arose during the trial of the perpetrator, and when books and films about them appeared that were not always easy to comprehend. The rough and direct humor characteristic of Northern Norway represented a cultural resource that had developed through centuries of harsh living conditions, and it proved unexpectedly valuable in this context.

Participants described that what initially seemed uncertain and difficult gradually became easier to handle because the survivors strengthened each other. Participants described that being together in the group reduced feelings of loneliness and helped them find a way forward in life. They were proud and grateful, and expressed compassion for survivors in other parts of the country who did not have the same opportunity.

### Organization and responsibility over time

Collective traumas such as acts of terror affect people differently than single events because those affected experience a strong fear that it may happen again, since the intention is deliberately to cause harm [23]. At the same time, there is often intense media attention, which is distressing and prevents them from finding peace. An additional problem is that survivors are often geographically dispersed, causing them to lose contact with each other. These challenges require the healthcare system to take overall responsibility and ensure that support is maintained over time [23, 27].

When examining responses to various terror events, help in the acute phase is usually satisfactory, whereas long-term follow-up often fails. This is due both to underestimating how long it can take to overcome fear and trauma reactions, and to not accounting for the fact that problems may reappear later or emerge at a later stage. Other factors include unclear responsibilities and lack of coordination. Research shows that insufficient duration of follow-up after terror is a recurring problem, and that long-term and coordinated efforts are necessary [5, 23, 27].

### Community, participation, and peer support groups

It is evident that terror attacks are a serious burden for the individuals affected and that they may weaken social bonds in society as a whole [23, 28]. The survivors experienced loss of friends and significant psychological and physical consequences. Therefore, psychosocial follow-up and support after a terror attack should be a central component of responsible psychosocial care following terror [9, 10]. Despite this widely accepted recognition, follow-up for survivors and bereaved individuals varies greatly over time in several countries [5, 8, 27].

It is striking that across studies, survivors and bereaved individuals express strong support for peer support groups [29–31]. Even those who do not participate themselves find it reassuring to know that the group exists as an option. Over time, survivors discover that participation in peer support groups provides a sense of belonging and meaning that they cannot achieve in traditional treatment settings [29, 30].

Being together around a shared experience such as a terror attack means that they do not need to explain themselves to each other, because everyone has experienced the same thing. In peer groups, they therefore avoid the stigma of being a damaged victim to be pitied. This counteracts loneliness, isolation, and unhappiness—challenges known from other trauma contexts, such as Norwegian merchant sailors during World War II [32].

Through psychoeducation and shared reflection, the survivors in Tromsø learned to put their feelings into words, and by recognizing that others in the group felt the same way, they experienced that their reactions were normal. This also involved showing solidarity during difficult phases, such as anniversaries of the attack, and supporting each other with practical challenges related to finances, health, and legal matters.

A key element in Tromsø was the survivors’ strong involvement in their own development by contributing to shaping the group’s practice. Through shared social activities, space for humor and optimism emerged. Our experiences therefore show that psychoeducational-like groups should have an important role alongside other interventions in long-term follow-up after a terror attack. However, the findings reflect participants’ retrospective experiences and do not allow conclusions about the specific effects of the group or broader generalizations. Our findings reflect a specific case, and their relevance to other settings may depend on factors such as shared identity and how the group was organized over time.

### Limitations

This study has several limitations. First, the data are based on retrospective accounts collected many years after the group sessions, which may be affected by recall bias. The findings therefore reflect how participants currently make sense of their experiences, rather than providing direct evidence of effects during the original period.

Second, the dual role of the researchers as both clinicians and interviewers represents a potential source of bias. Although familiarity with the group provided contextual insight, it required careful attention to reflexivity, and the findings should be interpreted with this in mind.

Third, the sample may reflect self-selection bias, as participants were individuals who chose to re-engage with the group experience after many years. This may have resulted in a more positive sample, while the absence of non-participants, including possible dropouts or less satisfied members, limits the range of perspectives captured.

Fourth, the use of focus groups may also have influenced the findings. While the method allowed participants to interact, it may also have reproduced group dynamics such as shared narratives and reduced expression of disagreement. As participants were reunited the method may have reinforced common interpretations rather than capturing the full range of experiences.

Finally, participants’ experiences cannot be understood independently of other forms of follow-up and life circumstances over time. Several had received individual therapy and other support, and some had experienced severe physical injuries. As these factors were not systematically examined, it is not possible to isolate the specific contribution of the group. Rather, the group should be understood as one component within a broader context of recovery.

In sum, these limitations may have influenced how participants described the group. The setting itself, including the presence of former leaders, may also have reinforced shared interpretations. The findings should therefore be interpreted with caution.

### Implications

We believe our study has some important implications. While the findings show how participants experienced this group, they do not in themselves determine how peer-support groups should be designed or used in other contexts. Following an incident, there is a need to get to know each individual and their needs for assistance and develop both short-term and long- term plans for follow-up. For the individual, it is important to quickly identify the type of help they need and what suits them best. One should designate responsible staff who can cooperate across organizational structures. Both individual and collective measures (peer support groups) should be provided, in addition to family-oriented arrangements, and sufficient funding should be secured. For the group work, a holistic approach, including other aspects in addition to health, could be beneficial. Monitoring the process and securing records and feedback from those affected are central elements in connected research.

## Conclusions

The findings suggest that psychoeducational-like groups may be experienced as a meaningful complement to other forms of support. Our findings reflect participants’ experiences of this group and can not be understood as general recommendations for all settings. How the various parts of the follow-up best can work together remain to be further explored.

Participants emphasized the importance of early initiation and sustained follow-up over time. In line with the findings of McMillan and Chavis [19], group identity and a mutual sense of relationship and safety among members seem to be central factors in the functioning of the group. Additionally, it is significant that participation and real influence are encouraged, and that members feel strengthened by being part of the group. Early intervention makes it possible to rapidly identify problems both at the group level and for individuals.

A central premise of the follow-up work was that the survivors already knew one another and had shared the attack, escape, and aftermath together, making community crucial from the very beginning. At the same time, the young survivors emphasized the importance of responsible adult leadership and a structured framework. While individual and family therapies addressed complex trauma reactions, the group gatherings offered a complementary arena where shared understanding, reflection, and humor could coexist in a calmer and more supportive atmosphere. Along the way, it is important for individuals to recognize their life goals and become aware of how these can be realized.

## Supplementary Information

Supplementary Material 1.

## Data Availability

The datasets generated during the current study are not publicly available due privacy reasons.

## References

[1] The 22 July C. The 22 of July 2011: Timeline Available from: https://www.22julisenteret.no/en/read-more/timeline.

[2] Store norske l. Terrorangrepene i Norge 22. juli 2011 Available from: https://snl.no/terrorangrepene_i_Norge_22._juli_2011.

[3] Norwegian Official Reports. Report of the 22 July Commission. NOU 2012:14. Oslo: Office of the Prime Minister; 2012. Available from: https://www.regjeringen.no/en/documents/nou-2012-14/id697260/.

[4] Dyregrov A, Dyregrov K, Straume M, Bugge RG. Weekend family gatherings for bereaved after the terror killings in Norway in 2011. Scandinavian Psychol. 2014;1:e8.

[5] Lereim I, Prietz R, Strand M, Klinkenberg E, Ellefsen M, Misvær G, et al. Læring for bedre beredskap. Helseinnsatsen etter terrorhendelsene 22. juli 2011. Oslo: Norwegian Directorate of Health; 2012.

[6] Mathiesen E. Assemblies of survivors from Utøya. Tidsskrift Den norske legeforening. 2015;135:108–9. 10.4045/tidsskr.14.1245 25625983

[7] Retriever. Mediedekning etter 22. juli. Oslo: Fritt Ord; 2022.

[8] Watkins J. The value of peer support groups following terrorism: reflections following the September 11 and Paris attacks. Australian J Emerg Manage. 2017;32:35–9.

[9] Eyre A. The value of peer support groups following disaster: from Aberfan to Manchester. Bereave Care. 2019;38:115–21.

[10] Eyre A. The manchester attack support group programme: modelling a psychosocial response to collective trauma. J Psychosocial Stud. 2022;15:16–35.

[11] Turunen T, Haravuori H, Pihlajamäki JJ, Marttunen M, Punamäki R-L. Framework of the outreach after a school shooting and the students perceptions of the provided support. Eur J Psychotraumatology. 2014;5:23079. 10.3402/ejpt.v5.23079. 10.3402/ejpt.v5.23079 PMC4082198 25018862

[12] Turunen T, Punamäki R-L. Professionally led peer support group process after the school shooting in Finland: Organization, group work, and recovery phases. Omega: J Death Dying. 2016;73:42–69.

[13] Forbes D, Creamer M, Phelps A, Bryant R, McFarlane A, Devilly GJ, et al. Australian guidelines for the treatment of adults with acute stress disorder and post-traumatic stress disorder. Aust N Z J Psychiatry. 2007;41:637–48. 17620160 10.1080/00048670701449161

[14] Tedeschi RG, Calhoun LG. Posttraumatic growth: conceptual foundations and empirical evidence. Psychol Inq. 2004;15(1):1–18.

[15] Drury J. Collective resilience in mass emergencies and disasters: a social identity model. In: Jetten J, Haslam C, Haslam SA, editors. The Social Cure: Identity, Health and Well-Being. Hove, UK: Psychology; 2012. pp. 195–215.

[16] Bonanno GA. Loss, trauma, and human resilience: have we underestimated the human capacity to thrive after extremely aversive events? Am Psychol. 2004;59(1):20–8. 14736317 10.1037/0003-066X.59.1.20

[17] Hobfoll SE, Watson P, Bell CC, Bryant RA, Brymer MJ, Friedman MJ. Five essential elements of immediate and mid-term mass trauma intervention: empirical evidence. Psychiatry. 2007;70:283–315. 18181708 10.1521/psyc.2007.70.4.283

[18] Charmaz K. Constructing Grounded Theory. 2 ed. London: Sage; 2014.

[19] McMillan DW, Chavis DM. Sense of community: a definition and theory. J Community Psychol. 1986;14:6–23.

[20] Jetten J, Haslam SA, Haslam C. The case for a social identity analysis of health and well-being. The social cure: Psychology; 2012. pp. 3–19.

[21] Glad KA, Dyb G, Boelen PA, Wentzel-Larsen T, Stensland SØ. Early predictors of prolonged grief among bereaved trauma survivors 8.5 years after a terrorist attack. Psychol Trauma. 2025;17:477–84. 38900511 10.1037/tra0001684

[22] Nilsen LG, Stene LE, Glad KA, Hafstad GS, Dyb G. Oppfølgingen av de som overlevde på Utøya. Oslo: NKVTS; 2017.

[23] Barker A, Dinisman T. Meeting the needs of survivors and families bereaved through terrorism. London: Victim Support; 2016.

[24] Antonovsky A, Health. Stress and Coping. San Francisco: Jossey-Bass; 1979.

[25] Borinca I, Andrighetto L, Valsecchi G, Berent J. Ingroup norms shape understanding of outgroup prosocial behaviors. Group Processes Intergroup Relations. 2022;25(4):1084–106.

[26] Borinca I, Sainz M, Gkinopoulos T. Social norms and peace. Peace and Conflict. J Peace Psychol. 2024;30(3):277.

[27] Reifels L, Pietrantoni L, Prati G, Kim Y, Kilpatrick DG, Dyb G. Lessons learned about psychosocial responses to disaster and mass trauma: an international perspective. Eur J Psychotraumatology. 2013;4:22897. 10.3402/ejpt.v4i0.22897. 10.3402/ejpt.v4i0.22897 PMC3873118 24371515

[28] Brady K, Randrianarisoa A, Richardson J. Best practice guidelines: supporting communities before, during and after collective trauma events. Carlton, Victoria: Australian Red Cross; 2018.

[29] Rew N. Supporting the survivors: experiences and perceptions of peer-support offered to UK-terrorist survivors. Int Rev Victimology. 2021;27:63–79.

[30] Repper J, Carter T. A review of the literature on peer support in mental health services. J Mental Health. 2011;20:392–411. 10.3109/09638237.2011.583947 21770786

[31] Wynn R. Psykiske plager hos veteraner. Behandling og støtte for personer med posttraumatisk stress. Oslo: Cappelen Damm; 2023.

[32] Hjeltnes G. Handelsflåten i krig 1939–1945. Bind 4: Krigsseiler: krig, hjemkomst, opprør. Oslo: Grandal og Dreyer; 1997.

