# Supplementary material for: The value of peer-support after the 22 July 2011 terrorattack: a follow-up focus-group study of gatherings for survivors from Utøya in Tromsø, Norway
Source: BMC Psychol. 2026 Jun 4;14:1150. doi: 10.1186/s40359-026-04921-8 (PMC13449565; doi:10.1186/s40359-026-04921-8)
Supplement: Supplementary file 1 [file 40359_2026_4921_MOESM1_ESM.pdf]

Interview guide

1. The significance of community

- What significance did the sense of community during the gatherings have for belonging, support, and coping in life after Utøya?
- How important was it to know that you were missed if you did not attend?
- What are your thoughts on the social activities outside the meetings themselves?

2. The significance of participation in shaping group practice

- What influence did you have on the design of the support provided?
- How did you experience the way the support was organized?

3. The significance of participating in the group over time

- Has the health support provided from 2011–2014 had an impact on your life in the years since?
- Did the group-based support last long enough?
- Did it have any impact on your everyday life that the collective support ended?

4. Other support that had significance

- For those who received both individual and group-based support: What were their respective strengths and weaknesses, and how did they complement each other?
- Did you receive concrete help with your problems, for example with reminders?
- Have you been offered support if/when you needed it in the years after 2014?

5. Space for emotions

- What are your thoughts on the gatherings having both a social part and a part where relevant issues and problems were discussed?
- What do you think about the role of humor in this context?

6. The political dimension

- Should we have talked more about the political background of the terrorist attack?

7. Media handling

- What impact has attention from different media (social media, newspapers, TV, film, literature, theatre) had on you?

8. The significance of follow-up over time

- Did you experience that measures from the healthcare system helped make you feel safer and less alone?
- Is there any kind of follow-up you would like in the future beyond what exists today?
- What do you think is important to consider for future follow-up of people in similar situations?
- What are your thoughts on the 'peer support project' that was attempted afterwards?
